# Use of Saliva in Alternative to Serum Sampling to Monitor Biomarkers Modifications in Professional Soccer Players

**DOI:** 10.3389/fphys.2018.01828

**Published:** 2018-12-20

**Authors:** Vincenzo Cristian Francavilla, Francesco Vitale, Marcello Ciaccio, Tindaro Bongiovanni, Claudia Marotta, Rosalia Caldarella, Lorenzo Todaro, Maurizio Zarcone, Roberto Muratore, Chiara Bellia, Giuseppe Francavilla, Walter Mazzucco

**Affiliations:** ^1^School of Engineering, Architecture, and Motor Sciences, Kore University of Enna, Enna, Italy; ^2^Department of Science for Health Promotion and Mother to Child Care “G. D’Alessandro,” University of Palermo, Palermo, Italy; ^3^Department of Laboratory Medicine, “P. Giaccone” University Hospital, Palermo, Italy; ^4^Nutrition, Hydration & Body Composition Department, Parma Calcio 1913, Parma, Italy; ^5^Complex Unit of Laboratory Medicine, “P. Giaccone” University Hospital, Palermo, Italy; ^6^U.S. Città di Palermo Football Club, Palermo, Italy; ^7^Clinical Epidemiology and Cancer Registry Unit, “P. Giaccone” University Hospital, Palermo, Italy; ^8^Department of Surgical, Anatomical and Oncological Disciplines, University of Palermo, Palermo, Italy

**Keywords:** salivary cortisol, salivary testosterone, IgA, salivary and serum hormones correlation, salivary hormones variation, competitive sports, soccer players

## Abstract

We aimed to investigate the correlation between serum and salivary concentrations of steroid hormones and IgA, and the variation in concentrations of these biomarkers, across a soccer competitive season in a sample of players playing for an Italian major League team. Thirty-five elite male soccer players were recruited and assessed for salivary hormones (cortisol, testosterone, T/C‰ and DHEA-S) and IgA at three different time-points: (t_1_) after the pre-season period and 16 official matches played; (t_2_) after a winter break and three official matches played; (t_3_) 2 days after the final match of the championship and 19 matches played. Players were also tested for blood biomarkers (ser-C, ser-T, ser-T/C‰, ser-IgA, ACTH) at two detection times (t_1_ and t_3_). Blood samples were collected immediately after saliva sampling. The Spearman’s rank correlation was used to explore the correlation between blood and salivary concentrations of cortisol, free testosterone and IgA in the different time points. One-way ANOVA and permutation test were performed to explore changes by time of hormones and IgA concentrations over the competitive season. We documented a positive correlation between serum and saliva concentrations for Cortisol at t_1_ (+58.2%; *p*-value = 0.002) and t_3_ (+54.2%; *p*-value = 0.018) and for Testosterone at t_1_ (+42.0%; *p*-value = 0.033). Moreover, a positive variation was documented across the season (D = t_3_–t_1_) for Cortisol (D = +6.83; SEM = ±2.70; Var% = +37.6; *p*-value = 0.032), Testosterone (D = +0.33; SEM = ±0.07; Var% = +27.3; *p*-value = 0.002) and DHEA-S (D = +44.48; SEM = ±18.54; Var% = +82.0; *p*-value = 0.042), while a decrease of sal-T/C ratio and no variation in salivary IgA concentrations were reported. In conclusion, our findings support for experimental use of saliva samples to monitor steroid hormones modifications in professional soccer players across a competitive season.

## Introduction

Physical activity in soccer has been demonstrated to modify the hormone levels and the immune function (Greig et al., 2006; Doan et al., 2007; Mallo and Navarro, 2008; Moreira et al., 2013). Recent studies have speculated on the hypothesis to use saliva as an alternative to blood sampling for the assessment of hormonal and immune response to acute exercise or training (Neary et al., 2002; Granger et al., 2004; Paccotti et al., 2005; Moreira et al., 2009, 2014; Wood, 2009; Crewther et al., 2011; Owen et al., 2016), since correlation between serum and saliva concentration has been investigated for some hormones (Cadore et al., 2008; Papacosta and Nassis, 2011). In fact, being that the sampling procedures are non-invasive, salivary biomarkers collection eliminate the risk of needle-stick injuries, is stress-free and may not require a trained health professional. For these reasons, salivary sampling has become increasingly attractive in sports science throughout the years (Gatti and De Palo, 2011).

Among all hormones, cortisol (C) and testosterone (T) have been studied more in relation to exercise. In particular, salivary cortisol (sal-C), which is a representative marker of circulating free cortisol as well as a reliable measure of the hypothalamic-pituitary-adrenal axis adaptation to stress thanks to the adrenocorticotropic hormone (ACTH response), can be used as an indicator of the body’s stress response to physical challenge or to a psychological stressor (Viru and Viru, 2004; Banfi and Dolci, 2006; Papacosta and Nassis, 2011; Gaviglio and Cook, 2014). Again, salivary testosterone (sal-T) and its precursor dehydroepiandrosterone sulfate (DHEA-S) have also been related to physical performance (i.e., speed, power and strength) (Crewther et al., 2009; Vingren et al., 2010), whereas assessment of the salivary testosterone/cortisol ratio (sal-T/C) could indicate the anabolic/catabolic adaptations of training (Crewther et al., 2005).

In soccer it has been shown that sal-C concentration increased after a competitive match in female players (Haneishi et al., 2007), while no significant changes in male professional soccer players have been already reported (Moreira et al., 2009; Oliveira et al., 2009). Moreover, there is discordant evidence on the changes in sal-T concentrations with exercise as some studies documented an increase (Thorpe and Sunderland, 2012) while others reported a decrease in players salivary levels (Filaire et al., 2001).

Further, intense exercise can also affect immunoglobulin A (IgA) secretion and salivary IgA could identify a vulnerability to infections mediated by overtraining (Putlur et al., 2004). In particular, an immediate decrease in salivary IgA levels, usually recovering within 24 h post-exercise, have been shown in many sportive contexts characterized by acute bouts of prolonged strenuous exercise (Tomasi et al., 1982; McDowell, 1991), such as international soccer tournaments (Peñailillo et al., 2015) and competitive training matches (Moreira et al., 2009). However, the usefulness of determining salivary IgA after exercise still remains under debate due to the lack of robust data (Maya et al., 2016).

Overall, the uncertainty found in current literature of having a clear framework of salivary hormones modifications in relation to exercise, and with soccer in particular, requires more research, with a special focus on the simultaneous assessment of the different available salivary biomarkers, with particular regard to their trend over longer observational periods.

This study aimed to investigate (i) the correlation between serum and salivary steroid hormones and IgA concentrations and (ii) the variation in the concentrations of these biomarkers across a competitive season in a sample of players of an elite Italian soccer team.

## Materials and Methods

A multidisciplinary research team, made up of sports physicians, epidemiologists and a nutritionist conceived this observational pilot study. We recruited a convenience sample represented by 35 elite male soccer players of an Italian major league team (“Serie A”). Study aims were presented to the players and all of them were recruited in the study at the beginning of the competitive season of 2016. Players’ characteristics (age, weight, height, nationality, years in professional leagues) and current competitive season statistics (number of matches played, substitutions, minutes played during every official match, injuries, diseases) were collected in a standardized electronic form by the medical and sanitary staff.

The research protocol was approved by the ethics committee and all players gave their written informed consent to participate in the study.

### Saliva Sampling

**FIGURE 1:**
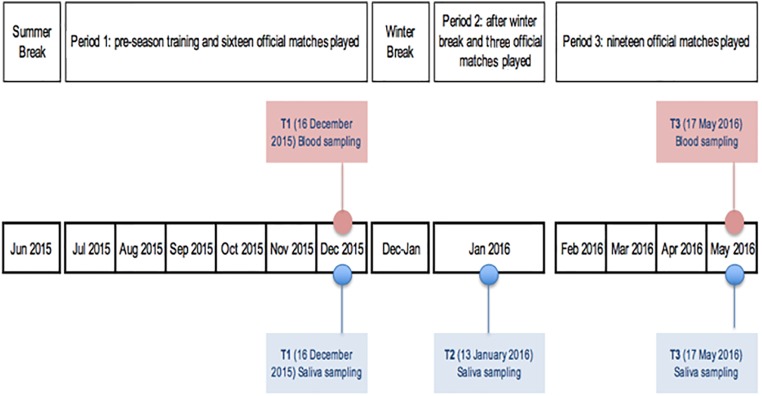
Salivary and blood sampling scheduled timeline.

The recruited players were assessed at three time points for salivary hormones in adherence to the following scheduled timeline (Figure 1): t_1_ (December 16, 2015): after the pre-season period and 16 official matches played; t_2_ (January 13, 2016): after winter break and 3 official matches played; t_3_ (May 17, 2016): 2 days after the final match of the championship and 19 official matches played (end of season).

Salivary samples were always collected prior to the blood ones and on Wednesday mornings, after the Sunday match, the Monday off and the first training of the week on Tuesday, by following the same standard conditions. Players were asked to maintain their normal diet and to refrain from alcohol as well as to consume water only (500 mL) to ensure adequate hydration and avoid variations in saliva secretion (Papacosta and Nassis, 2011; Thorpe and Sunderland, 2012). The subjects were also asked to abstain from food and caffeine products for at least 2 h prior to the collection of saliva. The recovery interval from the last bout of exercise was 24 h. Lastly, players were instructed not to perform any physical exercise and not to brush their teeth 2 h prior to the sampling to avoid micro-injuries or abrasion that could induce blood contamination of saliva.

Saliva was collected into Salivette© devices (Sarstedt, Germany) by sterile cotton rolls placed in the mouth. Players were required to rinse out their mouths with distilled water before starting the procedure. The saliva samples were collected while the players were in a seated position, with eyes open, head tilted slightly forward and making minimal orofacial movement (Walsh et al., 2004; Peñailillo et al., 2015). Players were asked to gently chew the swab for 3 min and, in any case, to keep it in their mouth until feeling that they can no longer prevent themselves from swallowing the saliva produced. Then, the saturated swab had to be returned to the suspended insert (pre-weighted vials) and the Salivette was firmly closed with the stopper (Lippi et al., 2009). Salivette© vials were stored in a fridge box and delivered to the laboratory where they centrifugated at 1500 × *g*/3 (min)–5 (max) minutes. The obtained liquid was volumetrically quantified and then transferred to low-binding polypropylene vials to be immediately frozen and stored at -20°C until assayed for cortisol, testosterone, IgA concentration and DHEA-S (Moreira et al., 2009). After de-frosting, the salivary samples were immediately analyzed.

Salivary cortisol in nmol/L, testosterone (sal-T) in nmol/L and DHEA-S in nmol/L were tested by electrochemiluminescence immunoassay with ECLIA (Roche Diagnostics S.p.a, Monza, Italy) integrated with Hitachi/Cobas e-411 (Roche Diagnostics S.p.a, Monza, Italy), while IgA (sal-IgA) in μg/mL were tested by immunoturbidimetric assay (Roche Diagnostics S.p.a, Monza, Italy) with Hitachi/Modular P (Roche Diagnostics S.p.a, Monza, Italy).

Salivary T/C ratio (sal-T/C) was converted in sal-T/C% in order to make the values readable.

### Blood Sampling

Blood hormones were taken within the scheduled routine lab examination. Blood collection followed the saliva sampling performed at t_1_ and t_3_ (Figure 1). After a 30-min rest in a comfortable seat, blood samples were collected via venipuncture from an antecubital arm vein using a safety butterfly set. Blood was collected into Vacutainer tubes containing SST-Gel and Clot Activator. The blood was allowed to clot at room temperature, and subsequently centrifuged (1500 *g*, 4°C, 15 min) for serum separation. The resulting serum was used for the measurements. The samples were stored and frozen at -80°C until analyzed. Serum Cortisol (ser-C), Testosterone (free and total form), and IgA (ser-IgA) were measured at two specific points during the season: t_1_ (16 December 2015) after the pre-season period and 16 official matches played and t_3_ (17 May 2016): 2 days after the final match of the championship and 19 official matches played. Ser-C in mcg/dL was analyzed with competitive immunoassay using direct chemiluminescence technology (Advia Centaur, Siemens), whereas free testosterone (ser-T) in pg/mL, the form used for the study, was analyzed with ELISA enzyme immunoassay, REF-EIA-29294 (DRG instruments GmbH, Germany) and ser-Ig-A in mg/dL was analyzed with Immunoturbidimetric assay on “KONELAB 600” instrument (SCLAVO Diagnostics International, Italy). ACTH in pg/mL was determined by using radioimmunoassay (RIA). Serum T/C ratio was converted in ser-T/C% in order to make the values readable.

### Statistical Analysis

A descriptive statistical analysis of the players’ characteristics and of steroid hormones concentrations tested in saliva (sal-C, sal-T, sal-IgA, DHEA-S) and blood (ser-C,ser-T, ser-IgA, ACTH) samples was performed. Salivary and serum T/C% were also described.

Mean ± standard deviation (SD) and extremes of ranges or mean ± standard error of the mean (SEM) were computed when appropriate.

The Spearman’s rank correlation (rho) was used, assuming mid-rank for ties, to explore the correlation between blood and salivary concentrations of cortisol, free testosterone and IgA in the different time points (t_1_, t_3_). Further, in order to identify the outliers for each measurement Chi-squared test, Dixon tests, Grubbs tests and box & whiskers method, drawn with a length of 1.5 times the interquartile range (data which lie beyond the extremes of the whiskers are considered potential outliers) were used.

One-way ANOVA with repeated measures was performed to explore changes by time of hormones and IgA concentrations over the competitive season in a restricted group of players whose salivary samples were available at each one of the three detection times. The Friedman’s test was used when the Shapiro–Wilk’s test showed failure of normality assumption. The Mauchly’s test of sphericity was performed to test the null hypothesis that the error covariance matrix of the orthonormalized–transformed dependent variable was proportional to an identity matrix. In case of violation of the assumption of sphericity, the significance was established by utilizing the Greenhouse–Geisser procedure. When assumptions were verified and a significant effect was found by ANOVA, a *post hoc* Tukey Honest Significant Difference (HSD) test was performed.

The permutation test was also performed to further analyse the variation by time across the season (Δ = t_3_-t_1_ and percentage of variation) in terms of paired differences between salivary biomarker concentrations. Variation percentages were also computed.

The significance level was set at *p* < 0.05. The data were analyzed using R version 3.3.2, released 2016/10/31 (R Core Team, 2016).

## Results

Table 1 reports the characteristics of the 35 major league team soccer players (42.9% Italians, 37.1% Europeans, and 20% extra-Europeans), including competitive season statistics and concentration levels of hormones and IgA sampled in blood and saliva, reported at the three different detection times. Average age of the players study group was 25.3 years (SD = ±4.9; min = 19, max = 37). The sample of players documented an average weight of 78.9 kg (SD = ±4.4; min = 67, max = 88) and an average height of 1.84 m (SD = ±0.05; min = 1.70, max = 1.97), while for BMI an average score of 23.3 (SD = ±0.8; min = 20.6, max = 25.4) was reported.

**Table 1:** Characteristics and competitive season statistics of the major League team soccer players recruited in the study and concentration levels of hormones and IgA in serum and salivary samples available at different detection times.

	Players		n. 35	
			
			mean (SD)	Min-Max
**Characteristics**	Age (years)		25.3 ( ± 4.9)	19–37
	Weight (Kg)		78.9 ( ± 4.4)	67–88
	Height (m)		1.84 ( ± 0.05)	1.70–1.97
	BMI (score)		23.3 ( ± 0.8)	20.6–25.4
	Years in professional leagues		7.77 ( ± 5.72)	1–23
	
	**Nationality**		**n. (%)**	
	
	Italian		15 (42.9)	
	European		13 (37.1)	
	Extra European		7 (20.0)	

**Competitive season statistic**	**Detection time**	**t_1_**	**t_2_**	**t_3_**

	n. of matches	16	3	19
	
		**mean (SD)**	**mean (SD)**	**mean (SD)**
	
	Minutes played	536 (±471)	107 (±113)	654 (±494)
	Substitutions (%)	10.3 (±14.4)	11.5 (±23.0)	11.4 (±13.7)
	Played matches per player (%)	47.2 (±35.9)	55.1 (±43.1)	53.2 (±32.9)

**Laboratory**		**t_1_**	**t_2_**	**t_3_**

**Saliva samples**	n. per time of sampling	26	26	19
	
	**Biomarker**	**mean (SD)**	**mean (SD)**	**mean (SD)**
	
	Cortisol (nmol/L)	18.6 (±6.8)	21.8 (±16.2)	25.3 (±7.0)
	Testosterone (nmol/L)	1.3 (±0.3)	1.2 (±0.4)	1.5 (±0.4)
	T/C ratio (‰)	77.3 (±37.9)	68.9 (±29.8)	66.3 (±34.4)
	DHEA-S (nmol/L)	44.7 (±28.1)	48.8 (±40.9)	97.6 (±84.5)
	IgA (μg/mL)	122.7 (±97.5)	131.9 (±98.0)	147.9 (±145.8)

**Serum samples**	n. per time of sampling	29	0	19
	
	**Biomarker**	**mean (SD)**	**mean (SD)**	**mean (SD)**
	
	Cortisol (nmol/L)	17.7 (±3.6)	–	18.4 (±3.4)
	Testosterone (pg/mL)	14.0 (±6.7)	–	12.4 (±6.0)
	T/C ratio (‰)	812.5 (±377.3)	–	689.6 (±330.7)
	ACTH (pg/mL)	45.1 (±21.7)	–	46.2 (±20.0)
	IgA (μg/mL)	165.1 (±52.9)	–	163.0 (±69.7)


*N., numbers; SD, Standard Deviation; Min, Minimum; Max, Maximum; T/C, ratio between blood free Testosterone (T) and blood Cortisol (C). T/C: ratio between salivary Testosterone and salivary Cortisol. DHEA-S, Dehydroepiandrosterone-Sulfate; IgA, Immunoglobulin A; ACTH, Adrenocorticotropic Hormone.*

The players’ competitive season statistics as well as available concentration levels of hormones and IgA in blood and salivary samples taken from the soccer players are presented at the three different detection times (Table 1).

On average, players played a higher number of minutes at t_1_ (536; SD = ±471) and t_3_ (654; SD = ±494) while they were substituted more frequently in the second (t_2_: 11.5; SD = ±23.0) and third (t_3_: 11.4; SD = ±13.7) parts of the season (Table 1).

The highest concentrations of C were detected at t_3_ both in salivary (n.19 players; mean = 25.30; SD = ±6.99; Min-Max = 15.89–42.76) and in serum (n.19 players; mean = 18.35; SD = ±3.43; Min-Max = 10.93–23.03) samples, with the highest T/C ratio (%) documented at t_1_ both for saliva (n.26 players; mean = 77.31; SD = ±37.90; Min-Max = 40–190) and serum (n.29 players; mean = 812.48; SD = ±377.26; Min-Max = 328–2265) (Table 1). Salivary T mean concentrations resulted of 1.25 (SD = ±0.31; Min-Max = 0.80–2.30) at t_1_ (n.26 players), 1.20 (SD = ±0.36; Min-Max = 0.70–2.10) at t_2_ (n.26 players) and 1.48 (SD = ±0.43; Min-Max = 1.00–2.70) at t_3_ (n.19 players) (Table 1).

The highest salivary DHEA-S mean concentrations were documented at t_3_ (n.19 players; mean = 97.63; SD = ±84.45; Min-Max = 6.76–312.04), while serum ACTH mean concentrations were 45.08 (SD = ±21.70; Min-Max = 16.37–110) at t_1_ (n.29 players) and 46.18 (SD = ±20.08; Min-Max = 14.7–77.12) at t_3_ (n.19 players) (Table 1).

Salivary IgA mean concentrations resulted in 122.69 (SD = ±97.51; Min-Max = 20–340) at t_1_ (n.26 players), 131.92 (SD = ±98.02; Min-Max = 20–410) at t_2_ (n.26 players) and 147.89 (SD = ±145.81; Min-Max = 20–570) at t_3_ (n.19 players), while serum IgA mean concentrations were of 165.07 (SD = ±52.87; Min-Max = 61.0–272.0) at t_1_ (n.29 players) and 163.00 (SD = ±69.72; Min-Max = 60.0–322.0) at t_3_ (n.19 players) (Table 1).

Samples used to explore any statistically significant correlation between blood and saliva hormones and IgA were available for 26 and 19 players at t_1_ and at t_3_, respectively (Table 2). A positive, significant correlation between blood and saliva samples was documented for C at t_1_ (+58.2%; *p*-value = 0.002) and at t_3_ (+54.2%; *p*-value = 0.018) and for T at t_1_ (+42.0%; *p*-value = 0.033), but a not significant correlation was found for T at t_3_ (+39.8%; *p*-value = 0.092) and for IgA at t_1_ (+13.3%; *p*-value = 0.518) and at t_3_ (-18.0%; *p*-value = 0.461) (Table 2). After excluding the outliers, a positive significant correlation between blood and saliva samples was confirmed for C at t_1_ (+50.6%; *p*-value = 0.013) and at t_3_ (+53.7%; *p*-value = 0.028) and was also documented for testosterone both at t_1_ (+61.8%; *p*-value = 0.003) and at t_3_ (+72.0%; *p*-value = 0.007), while a not significant correlation was confirmed for IgA at t_1_ (+14.8%; *p*-value = 0.547) and at t_3_ (+35.0%; *p*-value = 0.264).

**Table 2:** Concentration levels of hormones (cortisol, testosterone) and IgA in the soccer players of an Italian major league team: correlation between available serum and saliva samples (with and without outliers).

Biomarker	Time of sampling	N	Spearman’s Correlation rho (%)	*p*-value	N	Spearman’s Correlation rho^∗^ (%)	*p*-value
**Cortisolˆ**	**t_1_**	26	**+58.2**	**0.002**	24	**+50.6**	**0.013**
	**t_3_**	19	**+54.2**	**0.018**	17	**+53.7**	**0.028**
**Testosteroneˆˆ**	**t_1_**	26	**+42.0**	**0.033**	21	**+61.8**	**0.003**
	**t_3_**	19	+39.8	0.092	13	**+72.0**	**0.007**
**IgA°**	**t_1_**	26	+13.3	0.518	19	+14.8	0.547
	**t_3_**	19	-18.0	0.461	12	+35.0	0.264


*^∗^Analysis performed after excluding outlier.*

*ˆnmol/L.*

*ˆˆnmol/L for salivary samples and pmol/mL for blood samples.*

*°μg/mL.*

*IgA, Immunoglobulin A.*

In Table 3 is a summary of the results of the analysis restricted to n.11 players whose salivary biological samples were available for each one of three detection periods. The ANOVA analysis showed a statistically significant difference among detection periods for T (*p*-value = 0.047) and for DHEA-S (*p*-value = 0.014). Conversely, no statistically significant modification was highlighted at any detection time for C (*p*-value = 0.133), T/C ratio (*p*-value = 0.483) and IgA (*p*-value = 0.894). The *post-hoc* tests highlighted that the significant differences documented among periods were related to values between t_3_ (mean = 1.53; SEM = 0.11) and t_1_ (mean = 1.20; SEM = 0.07) for T (*p*-value = 0.041) and for DHEA-S in t_3_ (mean = 99.51; SEM = 24.74) in respect to t_1_ (54.67 ± 9.51, *p*-value = 0.027) and t_2_ (54.55 ± 12.68, *p*-value = 0.027) (Table 3).

**Table 3:** Concentration by detection times (t_1_, t_2_, t_3_) and its variation by time (Δ = t_3_–t_1_ and Var%) over the competitive season of salivary hormones (cortisol, testosterone, T/C ratio, DHEA-S) and IgA in 11 soccer players of an Italian major league team whose salivary samples were available at all detection times.

Salivary biomarkers	Time of sampling						
	
	t_1_	t_2_	t_3_	*p*-value^∗^	t_3_–t_1_^∗∗^		
					
Samples at detection time	n. 11	n. 11	n. 11		n. 11		
					
	Mean (SEM)	Mean (SEM)	Mean (SEM)		Δ (SEM)	Var%	*p*-value
**Cortisol (nmol/L)**	18.16 (±1.45)	21.90 (±4.30)	24.98 (±2.06)	0.133	**+6.83 (±2.70)**	**+37.6**	**0.032**
**Testosterone (nmol/L)**	*** 1.20 (±0.07)ˆ***	1.31 (±0.13)	*** 1.53 (±0.11)ˆ***	**0.047**	**+0.33 (±0.07)**	**+27.3**	**0.002**
**T/C ratio (‰)**	70.91 (±5.63)	66.36 (±6.91)	62.73 (±6.04)	0.483	-8.18 (±6.58)	-11.5	0.304
**DHEA-S (nmol/L)**	54.67 (±9.51)^$^	54.55 (±12.68)°	*** 99.51 (±24.74)***^$∘^	**0.014**	**+44.48 (±18.54)**	**+82.0**	**0.042**
**IgA (μg/mL)**	130.00 (±30.36)	140.91 (±31.69)	130.00 (±39.38)	0.894	0.00 (±28.95)	0	1.000


*^∗^1-way ANOVA repeated measure: One-way Analysis of Variance with repeated measures.*

*ˆTukey’s HSD *post-hoc* test: (t_3_–t_1_) p-value = 0.041.*

*^$^Tukey’s HSD *post-hoc* test: (t_3_–t_1_) *p*-value = 0.027.*

*°Tukey’s HSD *post-hoc* test: (t_3_–t_2_) *p*-value = 0.027.*

*^∗∗^paired sample tests.*

*SEM, Standard Error of the Mean; Δ, absolute difference among salivary values between t_3_ and t_1_; Var%, percentage variation between t_3_ and t_1_; T/C; ratio between Testosterone (T) and Cortisol (C); DHEA-S, Dehydroepiandrosterone-Sulfate; IgA, Immunoglobulin A.*

A positive statistically significant variation was documented across the season (D = t_3_–t_1_) for the paired differences in C (D = +6.83; SEM = ±2.70; Var% = +37.6; *p*-value = 0.032), T (D = +0.33; SEM = ±0.07; Var% = +27.3; *p*-value = 0.002) and DHEA-S (D = +44.48; SEM = ±18.54; Var% = +82.0; *p*-value = 0.042) (Table 3).

## Discussion

This observational study aimed to investigate the correlation between serum and salivary concentrations of steroid hormones and IgA as well as the variation across a competitive season in concentrations of these biomarkers in a sample of players playing for an Italian major League team (“Serie A”).

As psycho-physical activity in soccer is supposed to modify the hormone levels and the immune function (Greig et al., 2006; Doan et al., 2007; Mallo and Navarro, 2008; Moreira et al., 2013, 2014; Owen et al., 2016), previous studies investigated the importance to monitor serum biomarkers, particularly, cortisol, being an indicator of accumulated stress intensity (Engelmann et al., 2004), testosterone, considered as an index of body regeneration rate, and T/C ratio, proposed as an indicator for anabolic/catabolic balance and, therefore, of adaptation to training (Martínez et al., 2010). As blood sampling by using needle-stick is not stress free, recent studies speculated on the use of saliva samples as a useful alternative to blood sampling for the assessment of hormonal response to acute exercise or training (Neary et al., 2002; Granger et al., 2004; Paccotti et al., 2005; Cadore et al., 2008; Moreira et al., 2009; Wood, 2009; Crewther et al., 2011). Not by chance, the use of salivary non-invasive sampling procedures to monitor biomarkers concentrations has been of increasing interest in sports science due to it being a stress-free method. Moreover, avoiding the use of a needle-stick makes it easier to increase the sampling frequency, which could allow to better investigate the variation of biomarkers over time, potentially improving the management of professional players across a competitive season by the medical staff.

We documented a positive statistically significant correlation between serum and saliva concentrations for C and T at the two available detection times in a group of elite male soccer players both at the beginning and at the end of a competitive season. Moreover, the correlation resulted even stronger for T after excluding the outliers values, which could have been affected by individual factors related to specific events or conditions occurring in every single player across the competitive seasons.

The analysis restricted to the players whose salivary biological samples were available for each one of the three detection periods showed a positive statistically significant increase across the season in C and T, while the increase was even more consistent for DHEA-S. These results, whereas confirmed after a more frequent samples collection, could be of interest to interpret any potential biomarker variation in relation to relevant events characterizing a competitive season course.

The main limits of our study were represented by the small size study population and by the incomplete data for all of the recruited players at all detection times. The lack of completeness was due both to the players’ compliance and the typical seasonal variation in the composition of a professional soccer team, related to technical decisions, players’ incomings and outgoings, or to other factors including injuries or diseases occurring in players. Moreover, we have tried to explore the presence of any potential association between salivary steroid hormones’ concentrations and sports injuries, however, due to the small sample and the very limited number of injuries reported across the season, it was not possible to highlight any significant result.

Despite the discussed limitations, to the best of our knowledge, this is one of the few studies able to monitor and assess a team of professional soccer players by salivary sampling across an entire competitive major League. Particularly, the originality of our study is in the simultaneous reporting of modifications in salivary concentrations of C, T, DHEA-S, and IgA.

The main findings documented for the use of salivary sampling in our sample of elite soccer players can be summarized in: (1) a significant increase in sal-C concentrations across the competitive season, with a simultaneous weak increase of sal-T levels; (2) a decreasing of sal-T/C ratio during the season, which may reflect a greater psychological stress and an accumulated training and playing load, despite a not significant result; (3) a maintenance of salivary IgA concentrations during the championship, with a feeble not significant increase in the period characterized by a limited number of training sessions and of matches played.

Unlike other studies that reported a decrease in T concentration at the end of the competitive season (Filaire et al., 2001; Moreira et al., 2009; Arruda et al., 2015 Casanova et al., 2016), whereas saliva sampling was used to investigate the mood state and the performances in professional soccer teams, our study documented across the championship significant statistical modifications in salivary C, T, and DHEA-S levels for the restricted group of elite soccer players whose salivary samples were available at all detection times. Particularly, we observed a variation in terms of sal-C levels increasing, counterbalanced by an increase of sal-T levels, with a non-statistical decrease in T/C ratio.

Other studies testing professional soccer players by saliva sampling in pre- and post-matches, documented conflicting results as well. Peñailillo et al. (2015) assumed that a football match induces catabolic stress, as indicated by the decrease in T/C ratio, due to a decrease of salivary T concentrations, while Thorpe and Sunderland (2012) and Moreira et al. (2009) reported an increase in T concentration with no change in either C or testosterone to cortisol ratio, after a competitive football match. It was also revealed that T increases in response to resistance exercise (Cadore et al., 2008) and this response varies according to match outcomes and venues (Fothergill et al., 2017; Slimani et al., 2017). It has been previously described that testosterone increase in response to resistance exercise response vary according to the game outcome and venues.

Being that the reduction of T/C ratio reported by our study is a consequence of the simultaneous increase in C levels during a period of the season in which the team was involved in a fight to avoid relegation in the secondary league (Serie B), the documented variation may reflect the psychological mood of the players.

If, on one hand, the disagreement in findings on sal-T concentrations reported by the previous studies might be due to differences in samples, volumes and intensity of training, work-to-rest ratio and study design used, on the other hand, our results could lay the groundwork for adequate sportive training and good response to competitive matches as well as good performance capacity.

In accordance with previous studies (Collomp et al., 2015), we also monitored the seasonal variations of salivary DHEA-S concentrations in order to explore the adaptations to chronic physical exercise. We documented a consistent increase in salivary DHEA-S levels throughout the season and, in particular, a two-fold increase at the end of the season, probably in response to the recalled peak of stress activation, also documented by the increase in ACTH serum levels, used as an indicator of acute stress (Galbo, 1983; Carrasco and Van de Kar, 2003) so reflecting the asset of a team fighting to avoid a secondary league downgrade until the last match of the season.

The absence of a significant correlation between salivary and serum IgA concentrations documented in the study sample doesn’t seem to add further elements to speculate on the usefulness of this salivary biomarker after exercise, particularly on the potential vulnerability to infections associated with overtraining (Putlur et al., 2004; Maya et al., 2016). Nevertheless, the weak increase in salivary IgA concentrations – documented for the period of the season characterized by a limited number of training sessions, as compared to the periods of high intensity training characterized by an increase in T concentrations – suggests a possible role of T in limiting the IgA levels reduction, which is typically reported after intensive physical exercise: androgens, such as T, have been related to a protective effect over immunosuppression *in vitro* and animal models (Grossman, 1985). Previous studies exploring the variations of sal-IgA concentrations in football players showed heterogeneity and this may be due to different exercise intensities, time of the sample, large variability in IgA response between participants and psychological factors when training, simulated or official matches are used (Peñailillo et al., 2015). However, the results provided by our study do not add conclusive elements to the body of conflicting evidence regarding this protective effect of testosterone (Vingren et al., 2010).

The next steps for our future investigations will also include the study, by salivary sampling, of the variations in inflammatory or stress biomarkers with regard to athletes’ performances. Although the literature is limited, several inflammatory markers have been reliably determined from saliva and have increased significantly in response to stress (Slavish et al., 2015). Of interest, recent studies have reported that exercise training can deeply affect antioxidant defenses by inducing an excessive production of reactive oxygen or nitrogen species (RNS), so leading to oxidative stress-related tissue injury and impaired muscle contractility (Becatti et al., 2017; Mello et al., 2017) or fatigued states and underperformance (Lewis et al., 2015). The production of reactive oxygen and RNS is a fundamental feature of mammalian physiology, cellular respiration and cell signaling, and essential for muscle function and training adaptation. Aerobic and anaerobic exercise results in alterations in redox homeostasis (ARH) in untrained, trained and well trained. Low to moderate doses of and RNS play a role in muscle adaptation to endurance training, but an overwhelming in RNS and may lead to increased cell apoptosis and immunosuppression, fatigued states and underperformance Although the literature is limited, several inflammatory markers have been reliably determined from and have increased significantly in response to stress across multiple studies, with effect sizes ranging from very small to very large.

## Conclusion

In conclusion, our findings support for the experimental use of saliva samples to monitor the modification of specific biomarkers, such as steroid hormones, in professional soccer players across a competitive season.

Further studies on a more consistent number of players and with a higher frequency in data collection and biomarkers sampling should be performed in order to confirm the evidence provided by our study. These evidences could address future researches in order to predict psycho-physical effects in response to stressors as well as to investigate the effects of physical exercise and sport activities in professional soccer players. In the same direction, may be in a not distant future, it’ll possible to explore any potential association between salivary steroid hormones concentrations and sports injuries (Francavilla et al., 2016) or diseases affecting professional players (Francavilla et al., 2007).

## Ethics Statement

Ethical approval was provided by the “Palermo Ethical Committee 1” on April 14, 2018 (Protocol number: 04/2018).

## Author Contributions

All individuals listed as authors have contributed substantially to designing, performing or reporting the study and every specific contribution is indicated as follows. FV, TB, and WM: conception and design of the study. MZ, CM, and WM: statistical analysis. WM, VF, TB, and CB: interpretation of data. VF, WM, TB, MC, and RC: manuscript writing and drafting. VF, TB, WM, GF, MC, FV, RC, RM, and LT: revision of the manuscript. VF, FV,MC, TB, CM, RC, LT, MZ, RM, CB, GF, and WM: approval of the final version of the manuscript.

## Conflict of Interest Statement

LT and TB are employed by company U.S. Città di Palermo Football Club and by Parma Calcio 1913, respectively. The remaining authors declare that the research was conducted in the absence of any commercial or financial relationships that could be construed as a potential conflict of interest.
